# Sarcomas in hereditary retinoblastoma

**DOI:** 10.1186/2045-3329-2-15

**Published:** 2012-10-04

**Authors:** Ruth A Kleinerman, Sara J Schonfeld, Margaret A Tucker

**Affiliations:** 1 Epidemiology and Biostatistics Program Division of Cancer Epidemiology and Genetics, National Cancer Institute, National Institutes of Health, 6120 Executive Boulevard, Rockville, MD 20852, USA; 2 Human Genetics Program, Division of Cancer Epidemiology and Genetics, National Cancer Institute, National Institutes of Health, 6120 Executive Boulevard, Rockville, MD 20852, USA

**Keywords:** Retinoblastoma, Soft tissue sarcoma, Bone sarcoma, Radiotherapy, Epidemiology, *RB1* gene, Hereditary

## Abstract

Children diagnosed with the hereditary form of retinoblastoma (Rb), a rare eye cancer caused by a germline mutation in the *RB1* tumor suppressor gene, have excellent survival, but face an increased risk of bone and soft tissue sarcomas. This predisposition to sarcomas has been attributed to genetic susceptibility due to inactivation of the *RB1* gene as well as past radiotherapy for Rb. The majority of bone and soft tissue sarcomas among hereditary Rb survivors occur in the head, within the radiation field, but they also occur outside the radiation field. Sarcomas account for almost half of the second primary cancers in hereditary Rb survivors, but they are very rare following non-hereditary Rb. Sarcomas among hereditary Rb survivors arise at ages similar to the pattern of occurrence in the general population. There has been a trend over the past two decades to replace radiotherapy with chemotherapy and other focal therapies (laser or cryosurgery), and most recently, chemosurgery in order to reduce the incidence of sarcomas and other second cancers in Rb survivors. Given the excellent survival of most Rb patients treated in the past, it is important for survivors, their families and health care providers to be aware of the heightened risk for sarcomas in hereditary patients.

## Introduction

Children diagnosed with the hereditary form of retinoblastoma (Rb), a rare eye cancer caused by a germline mutation in the *RB1* tumor suppressor gene, have excellent survival, but face an increased risk for the development of sarcomas, both soft tissue (STS) and bone. This predisposition to sarcomas in retinoblastoma survivors has been attributed to genetic susceptibility as well as past radiation treatment for Rb.

### Retinoblastoma epidemiology

Retinoblastoma is a rare pediatric cancer of the eye with an autosomal dominant inheritance pattern. It is caused by mutations in the *RB1* tumor suppressor gene, located on chromosome *13q14* with very high penetrance and expressivity [1]. Approximately 80%-90% of *RB1* gene carriers develop ocular tumors. This gene encodes the cell cycle regulatory retinoblastoma gene protein (pRb), controls cellular differentiation during both embryogenesis and in adult tissues, regulates apoptotic cell death, maintains cell cycle arrest and preserves chromosome stability [2].

Retinoblastoma occurs in two forms: hereditary (30-40%) and non-hereditary (60-70%). Hereditary retinoblastoma is caused by a germline mutation in one allele of the *RB1* gene and an acquired somatic mutation in the other allele, whereas the non-hereditary form is caused by somatic mutations in both alleles. The hereditary form is characterized by disease in both eyes (bilateral Rb) and is typically diagnosed before 12 months of age, whereas, the non-hereditary form affects one eye (unilateral Rb) and is diagnosed between 2–5 years of age. About 10-15% of patients with unilateral Rb, however, carry a germline mutation and are considered hereditary. This difference in diagnosis age led Knudson to develop the two-hit theory [3], in which only one additional mutation is needed for hereditary Rb and two hits or somatic mutations are needed for non-hereditary Rb [4]. The age-adjusted annual incidence rate of retinoblastoma is 3.1 per 10^7^ with a 5-year relative survival of 97.5% in the U.S. [5]. Treatment for Rb has historically consisted primarily of radiotherapy (both external beam and radioactive plaques), enucleation, chemotherapy, focal therapies such as laser or cryotherapy, or a combination of these modalities.

### Subsequent malignancies after retinoblastoma

Long-term survivors of hereditary retinoblastoma are at an increased 20-fold risk of developing and dying from a subsequent non-ocular cancer, primarily bone and soft tissue sarcomas, melanoma and brain tumors [6,7]. Survivors of non-hereditary Rb are at much lower risk of a subsequent primary cancer, similar to the risk in the general population [8-10]. The risk for sarcomas in hereditary patients has been attributed to genetic susceptibility and past treatment with radiation [8,11,12]. In addition to radiotherapy, chemotherapy, specifically alkylating agents, has been associated with the risk of bone cancer after Rb [6,13,14], but less so for soft tissue sarcomas [15].

### Bone sarcomas after retinoblastoma

#### Patterns of risk

Bone sarcomas are one of the most common second primary cancers occurring after hereditary retinoblastoma accounting for 25%-30% of all second primary cancers [6-8,16,17]. Bone sarcomas are typically diagnosed in Rb survivors between 10 and 20 years of age, similar to the incidence pattern in the general population [5]. In these studies, the majority of bone sarcomas occurred within the radiation field in the head region, but up to 40% was diagnosed outside the treatment field, primarily in the lower legs [8,11,17].

Table 1 presents risks for bone sarcomas from epidemiologic cohort studies including at least 100 hereditary Rb survivors. The standardized incidence and mortality rates for bone sarcomas are increased several hundred-fold compared to population rates, due to the rarity of these tumors in the general population. A much lower risk for bone sarcomas was observed in the one cohort study that included non-irradiated survivors and began follow-up 25 years after Rb diagnosis [10]. It has been estimated that the cumulative incidence of bone sarcoma following retinoblastoma is 7% at 20 years [13,18]. Osteosarcoma is the most common type of bone sarcoma reported after Rb, but both chondrosarcoma and Ewing sarcoma have been reported as well [19,20], although risk estimates are not available for these other two types.

**Table 1:** Summary of bone sarcoma after retinoblastoma in cohort studies of 100 or more hereditary retinoblastoma survivors

**Study**	**Study design, Years of Rb diagnosis**	**No. subjects with hereditary retinoblastoma**	**Years of follow-up: median/mean**	**No. bone sarcoma cases**	**O/E, 95% CI**	**O/E by treatment for retinoblastoma**	**Comments**
**1a. Incidence**							
Kleinerman 2005 [7] US Two US medical centers	Hospital-based 1914-1984	963 1-yr survivors	Mean: 25	75	360 (283–451)	Any radiation: 406 (318–511) No radiation: 69 (8.4-250) Radiation + chemotherapy: 539 (384–733); Radiation, no chemotherapy: 302 (205–428)	AER = 29.6
Reulen 2011 [16] British Childhood Cancer Survivor Study, UK	Population-based 1940-1991	NA*, 5-yr survivors	Mean: 26	35	289 (209–402)	NA	AER = 23; * No. of Rb survivors not given but there are estimated to be 809 hereditary Rb subjects based on MacCarthy et al. [44]
Marees 2008 [6] Netherlands Dutch Retinoblastoma Registry	Registry -based 1945-2005	298 survivors	Median: 22	16	314 (180–511)	Radiation only: 302 (130–596) Radiation + chemotherapy: 586 (215–1275); Surgery only: 75 (1.9-421)	AER = 23
Tucker 1987 [14] US Late Effects Study Group	Hospital-based 1945-1979	319 2-yr survivors	Mean: 7	12	999 (515–1745)		*Hereditability not specified
**1b. Mortality**					**SMR, 95%CI**	**SMR by treatment for retinoblastoma**	
Yu 2009 [12] US Two medical centers	Hospital-based 1914-1996	1092 1-yr survivors	Median: 29	56	595 (449–773)	Radiation: 673 (506–879)	AER = 19.8; *No difference between males and females
Marees 2009 [46] Netherlands Dutch Retinoblastoma Registry	Registry-based 1862 - 2005	337 (alive in 1961)	Median: 26 yr Follow-up 1961-2005	11	289 (144–517)	Radiation only: 266 (72.2-680) Radiation + chemotherapy: 659 (179–1686); Surgery only: 124 (15–449)	Majority deaths from bone cancer occur within first 30 years
Acquaviva 2006 [46] Italian Retinoblastoma Registry	Registry-based 1923-2003	408	Median: 11	9	392 (204-753)	NA	
Fletcher 2004 [10] Patients from British hospitals and linkage with national registry	Hospital-based 1873-1950	144 25-yr survivors	Follow up began in 1940 Median age: 60	1	32.4 (0.82 - 180)	NA	*Radiation was not typically used to treat Rb during these years

Abbreviations: O = observed number of bone sarcomas; E = expected number of bone sarcomas; CI = confidence intervals; AER = absolute excess risk per 10,000 persons, yr = year; SMR = standardized mortality ratio; NA not available.

#### Treatment for Rb and risk of bone sarcomas

Both high-dose radiation and increasing cumulative dose of chemotherapy, mainly alkylating agents (cyclophosphamide and triethylenemelamine or TEM), have been linked to the occurrence of bone sarcomas following hereditary Rb [13,14]. Higher risks have been noted for the combination of radiotherapy and chemotherapy compared to either treatment alone [6-8,13,14]. An earlier study of British Rb patients provided some evidence that cyclophosphamide may increase the effect of radiotherapy on the risk of bone sarcoma [8].

In a case–control study of bone and soft tissue sarcomas after hereditary Rb, risk increased with increasing dose up to 10.7-fold at doses greater than 60 Gy [11]. The mean dose to the head among cases was 32.8 Gy, whereas the lower limbs had received virtually no radiation (<0.1 Gy). In an update of that study, the location of 75 bone sarcomas was skull and face (61%), lower limbs (29%), trunk (7.6%), and unknown location (3.8%) [7].

Based on a series of 155 osteosarcomas following hereditary Rb identified from the literature and one institute, investigators reported that the mean age of onset was related to the osteosarcoma location [21]. Sarcomas occurring in the radiation field were diagnosed one year earlier compared to those diagnosed outside the field (mean age = 12.2 years [range 3–35] vs. mean age = 13.4 years [range 4–22]. This age difference suggested to the investigators that different biologic mechanisms may be associated with the development of bone sarcomas depending upon the location in the body.

Studies of other pediatric malignancies have also reported an increased risk for second osteosarcomas following radiation and chemotherapy treatment for a first cancer (for a detailed review of radiation-related sarcomas, see Berrington de Gonzalez et al. in this issue).

### Soft Tissue Sarcomas

#### Patterns of risk

Soft tissue sarcomas (STS) are also one of the most common subsequent cancers following hereditary Rb accounting for 12% up to 32% of all second cancers [6,7,16]. In one large cohort study, an increased risk for STS was first observed within 10 years of Rb diagnosis and continued through adult life up to 50 years after Rb, with specific subtypes occurring at similar ages as in the general population [22,23]. Fifty years after radiation treatment for hereditary Rb, the cumulative risk of developing a STS was 13.1%, and the cumulative incidence for a STS inside the radiation field was higher than outside the field (8.9% vs. 5.1%) [22]. Table 2 presents the incidence and mortality due to STS after Rb in cohort studies of at least 100 hereditary Rb survivors.

**Table 2:** Summary of soft tissue sarcoma after retinoblastoma in cohort studies of 100 or more hereditary retinoblastoma survivors

**Study**	**Study Design Years of Rb diagnosis**	**Number of subjects with hereditary retinoblastoma**	**Years of follow-up: median/mean **	**No. of Soft tissue sarcomas**	**O/E, 95% CI**	**O/E by treatment for retinoblastoma**	**Comments**
**2a. Incidence**							
Kleinerman 2007 [22] US Two medical centers	Hospital-based 1914-1984	963 1-yr survivors	Mean: 25	69	184 (143–233)	Any radiation: 212 (164–270); No radiation: 47 (9.4-137); Any chemotherapy: 236 (161–333); No chemotherapy: 193 (133–271)	AER = 27 *No evidence of risk modification by sex *SIRs highest within first 10 years but remained significantly elevated ≥30
Reulen 2011 [16] British Childhood Cancer Survivor Study	Population-based 1940-1991	NA, 5-yr survivors	Mean: 26	16	N/A	N/A	Rates increase over time since Rb (highest >25)
Marees 2008 [6] Netherlands Dutch Retinoblastoma Registry	Registry-based 1945-2005	298	Median: 22	20	243 (148–375)	Radiation only: 303 (161–517) Radiation + chemotherapy: 354 (129–770) Surgery only: 48.4 (1.23-270)	AER = 29; SIRs elevated in all time periods (3 cases ≥40)
Tucker 1987 [14] US Late Effects Study Group	Hospital-based 1945-1979	319 2-yr survivors (hereditability not specified)	Mean: 7	4	235 (64–602)		All cases observed among females
**2b. Mortality**					**SMR, 95% CI**	**SMR by treatment for retinoblastoma**	
Yu 2009 [12] US Two medical centers	Hospital-based 1914-1996	1092 1-yr survivors	Median: 29	31	329 (223–467)	Any Radiation 395 (268–560)	AER = 10.9; SMR is higher for women vs men (not statistically significant)
Marees 2009 [45] Netherlands Dutch Retinoblastoma Registry	Registry-based 1862 - 2005	337	Median: 26 Follow-up 1961-2005	13	276 (147–472)	Radiation only: 311 (101–725) Rad + chemotherapy: 940 (345–2064); Surgery only: 85.2 (10.3-308)	*Deaths observed up to ≥50 years after RB *SMR peaks at 20–29 years but SMRs significantly elevated in all time periods
Acquaviva 2006 [46] Italian Retinoblastoma Registry	Registry-based 1923-2003	408	Median: 12	6	453 (203.5 - 1008)	NA	
Fletcher 2004 [10] UK Patients from British hospitals; linkage with national registry	Hospital-based 1873-1950	144 25-yr survivors	Median attained age: 60; Follow-up began in 1940	4	110 (29–281)	NA	*Treatment not available, but radiation was not typically used during these years of Rb diagnosis

Abbreviations: O = observed number of soft tissue sarcomas; E = expected number of soft tissue sarcomas; CI = confidence intervals; AER = absolute excess risk per 10,000 persons, yr = year; SMR = standardized mortality ratio; NA not available.

#### Subtype heterogeneity

STS diagnosed in Rb patients comprise a heterogeneous group of tumors of fat, cartilage and muscle; however, only one study has evaluated the risk of STS by histology after hereditary Rb [22]. Leiomyosarcoma (LMS) constituted the most common type of STS after Rb, with the majority diagnosed 30 and more years after Rb. This is consistent with LMS being one of the most common STS in the general population [23]. Although many LMS occurred in the head and neck region, the majority of LMS in females were diagnosed in the uterus [24]. Loss of heterozygosity in *RB1* has been reported in uterine LMS [25], which may confer an increased susceptibility to this tumor in this population. LMS of other pelvic sites have also been reported after Rb [26], and there have been several case reports of LMS diagnosed in the bladder [27,28].

Very high risks have also been observed for fibrosarcomas, rhabdomyosarcomas and pleomorphic sarcomas within the first 10 years after Rb [22,29]. These histologic types comprised the majority of STS that were diagnosed in or near the field of radiation, in contrast to LMS, which were more likely to occur outside the radiation field (Table 3). Only 10% of rhabdomyosarcomas arise in the soft tissue of the head, neck or face in the general population, whereas all of the rhabdomyosarcomas arose in the head following radiation for Rb [22].

**Table 3:** Location of soft tissue sarcoma after radiotherapy for retinoblastoma*

**STS subtype**	**In-field**	**Out-of-field**	**Total**
Leiomyosarcoma	8 (38.1)	13 (61.9)	21 (100.)
Fibrosarcoma	13 (100.)	0	13 (100.)
Pleomorphic sarcoma	11 (100.)	0	11 (100.)
Rhabdomyosarcoma	7 (100.)	0	7 (100.)
Liposarcoma	1 (33.3)	2 (66.7)	3 (100.)
Other STS	8 (80.0)	2 (20.0)	10 (100.)
Total	48 (72.7)	18 (27.3)	66 (100.)

*Based on data from Kleinerman et al. [22].

An increased risk for liposarcomas that began 10 years after diagnosis of hereditary Rb was observed in the study by Kleinerman et al. [22]. Lipomas, a benign tumor of fat tissue, have also been reported to be increased in that cohort, and the investigators noted a possible association between lipomas and subsequent risk of a soft tissue sarcoma [30]. Following this observation, a *RB1* mutation was identified in lipomas from hereditary Rb patients [31,32].

It has been suggested that females may be at higher risk of STS after hereditary Rb [9], but studies of Rb survivors have not consistently reported a higher risk among females. Males have a higher rate of Rb in the general population and all liposarcomas and lipomas occurred in males in the cohort in which they were evaluated [22,30].

#### Treatment for Rb and risk of STS

Although both radiotherapy and chemotherapy for hereditary Rb have been associated with an increased risk for STS, the evidence is more consistent for radiotherapy. (For a detailed review of radiation-related sarcoma, see Berrington de Gonzalez et al. in this issue). Wong et al. demonstrated a radiation dose–response for STS whereby risk increased with dose up to a significant 11-fold increased risk at ≥60 Gy [11]. The risk for STS was not associated with increasing alkylating agent score in the same cohort [22], whereas in another study of STS after all types of pediatric malignancies, including Rb, the risk for STS increased significantly with cumulative dose of alkylating agents, adjusted for radiation exposure [15]. Increased risks of STS have also been noted following surgery only for hereditary Rb [6,10].

#### Molecular evidence for an association of sarcomas with RB1

In additional to the epidemiologic evidence of an excess risk for both bone and STS in hereditary Rb patients, structural alterations of the *RB1* gene are well documented in primary bone sarcomas [33] and soft tissue sarcomas [34-36]. Most of the bone and soft tissue sarcomas diagnosed in hereditary Rb patients have complex karyotypes, including fibrosarcoma, LMS, pleomorphic sarcoma, liposarcoma and osteosarcoma that are all related to inherited defects in the RB pathway [37]. A comprehensive review by Burkhart and Sage of cellular mechanisms of tumor suppression by the retinoblastoma gene discusses the loss of *RB1* function and cancer progression [2].

## Conclusion

Hereditary Rb patients are at significant risk of developing a sarcoma due to past radiation treatment and genetic susceptibility. Sarcomas account for approximately 40% to 60% of second cancers in hereditary Rb survivors. There is convincing epidemiologic evidence linking past radiotherapy with sarcomas in hereditary patients. Risk of bone and STS begins within 10 years of treatment for hereditary Rb and continues throughout adulthood, most notably for STS.

Recognition of the increased risk for sarcomas associated with past radiotherapy has influenced the current treatment of retinoblastoma with a trend towards greater use of chemotherapy, focal therapies, and most recently, chemosurgery [38-40]. In addition, guidelines for imaging children for pre-treatment diagnostic evaluation of Rb without the use of ionizing radiation have been recommended to reduce the risk of second cancers in Rb patients [41]. However, the risk for bone sarcomas and STS remains, reflecting the genetic predisposition to these sarcomas due to loss of heterozygosity in the *RB1* gene. Patients who were treated in 1960s and 1970s with radiotherapy are still at risk in their adult years for the development of STS. Given the excellent survival of most retinoblastoma patients, it is important for survivors, their families and health care providers to be aware of these risks, especially for hereditary patients [42]. There is on-going research to try to identify whether specific *RB1* mutations or location of mutations predispose to sarcomas, which could lead to identification of those survivors at greatest risk [43]. The development of comprehensive guidelines for long-term follow-up that are specifically tailored for detection of sarcomas and other second primary cancers in retinoblastoma survivors are also needed, especially for those patients who received radiotherapy in the past.

## Abbreviations

Rb: retinoblastoma; STS: soft tissue sarcoma; LMS: leiomyosarcoma.

## Competing interests

The authors declare that they have no competing interests.

## Author contributions

RK and SS participated in the review of existing data, RK, SS and MT contributed to the interpretation of the data, and all participated in the draft of the manuscript. All authors read and approved the final manuscript.
